# Ring finger sensory latency difference in the diagnosis and treatment of carpal tunnel syndrome

**DOI:** 10.1186/s12883-021-02462-8

**Published:** 2021-11-05

**Authors:** Qingping Wang, Hong Chu, Hongyang Wang, Yan Jin, Xiaoquan Zhao, Chao Weng, Zuneng Lu

**Affiliations:** Department of Neurology, Renmin Hospital of Wuhan University, Wuhan, 430060 Hubei Province PR China

**Keywords:** carpal tunnel syndrome, median nerve, ulnar nerve, nerve conduction studies, sensory distal latency

## Abstract

**Objective:**

To explore the sensitivity of median and ulnar nerve sensory latency differences in diagnosing carpal tunnel syndrome (CTS) at different severities.

**Methods:**

CTS patients were divided into three groups based on disease severity (mild, moderate, and severe). Distal latency of sensory nerve action potential (SNAP) for the median and ulnar nerves was recorded. The sensitivity of SNAP distal latency to CTS and its correlation with CTS severity were analyzed.

**Results:**

Significant differences were found in the median nerve sensory action potential distal latency (MSDL) and in the median and ulnar sensory latency difference to ring finger (MUD) but not in the ulnar nerve sensory action potential distal latency (USDL) between CTS and control. The sensitivity and specificity were 92.2 and 99.4% with an MSDL cutoff value of 2.40 ms, respectively, and were both 100% with a MUD cutoff value of 0.33 ms. There was no significant difference in USDL among the CTS and control groups. Significant differences were found in MSDL and MUD among the CTS severities and between mild and moderate CTS, but not between mild and severe CTS or between moderate and severe CTS. Correlations with CTS severity were observed for MSDL and MUD but not for USDL.

**Conclusion:**

The ulnar nerve of the CTS patients was not damaged. A smaller MSDL reflected median nerve damage, which can be used for the early diagnosis of CTS. MUD correlated with CTS severity with a higher sensitivity than MSDL, which can provide therapeutic insight without pain to patients.

## Introduction

Carpal tunnel syndrome (CTS), the most common and widely studied nerve entrapment syndrome in the upper extremity, is caused by compression of the median nerve (MN) at the wrist as it passes through a space-limited osteofibrous canal. CTS is characterized by symptoms of pain and paresthesia in the hand and can involve the forearm, the upper arm, and even the shoulder in severe cases. Initially, patients with CTS often experience intermittent nocturnal paresthesia and sensory disturbances that can increase in occurrence frequency during waking hours. Subsequently, a loss of sensation develops, along with weakness and thenar muscle atrophy in later disease stages [1]. Diagnosis of CTS is based on clinical symptoms and the findings from physical examination and electrodiagnostic (EDX) tests, primarily in nerve conduction studies (NCS) [2]. However, studies have shown that routine EDX tests have limited sensitivity and specificity for the diagnosis of CTS [2–4]. Therefore, the American Association of Electrodiagnostic Medicine (AAEM) proposed the use of the median sensory nerve conduction and a comparison of the median and ulnar sensory nerve conduction, which have high sensitivity for the diagnosis of CTS [5].

Conservative and non-surgical options are recommended in the early disease stage, including oral medications such as non-steroidal anti-inflammatory drugs (NSAIDs), resting wrist splint, physical agent modalities, and local injections including corticosteroid and platelet-rich plasma (PRP) [6–8]. Surgical release of the retinaculum has been approved for moderate to severe CTS [9]. Studies have found that patients with mild symptoms tend to postpone medical treatment until the development and worsening of numbness and thenar atrophy, and patients with severe symptoms often have a slow recovery even after surgery [10].

The objective of this study was to compare the sensitivity and specificity of different neuroelectrophysiological indexes in the diagnosis of CTS. For the severity classification of CTS, the values of the median nerve sensory action potential distal latency (MSDL) and the median and ulnar sensory latency difference to ring finger (MUD) were evaluated with the aim of diagnosing and classifying CTS early and conveniently in order to guide patient treatment.

## Methods

### Patient Enrolment

From July 2019 to January 2021, eligible patients were diagnosed with CTS in the Department of Neurology of Renmin Hospital of Wuhan University based on clinical manifestations that met the CTS diagnostic criteria described by Pugdahl et al. [11]. Patients with wrist trauma and deformity, polyneuropathy or radiculopathy, and acute or chronic demyelinating disease were excluded through electrophysiological diagnosis. Electrophysiological data for the healthy wrists of the same patients with CTS on one hand were recorded for the control group.

### Nerve Conduction Studies (NCS)

All patients underwent NCS with the Keypoint Workstation (31A06, Alpine BioMedApS, Denmark). The room temperature was kept between 25 °C and 28 °C to maintain a consistent skin temperature between 32.0 °C and 34.0 °C in the patients’ hands. We recorded the antidromic sensory nerve action potential and the peak latency. Filters were set between 20 Hz and 3 kHz. The severity of CTS was classified as mild, moderate, and severe based on the NCS results (Table 1).

**Table 1:** Diagnostic criteria for the severity of CTS

Severity	Criteria
Mild	Prolonged sensory latencies ± SNAP amplitude below the lower limit of the normal value with normal motor studies
Moderate	Prolonged median motor distal latency in addition to abnormal sensory latencies as noted for mild CTS
Severe	Any of the aforementioned NCS abnormalities with evidence of axonal loss defined by either: (1) an absent or low amplitude SNAP; (2) a low amplitude or absent thenar CMAP; or (3) a needle electromyogram revealing fibrillation potentials or neurogenic motor unit changes

*CMAP* Compound muscle action potential, *CTS* Carpal tunnel syndrome, *NCS* Nerve conduction studies, *SNAP* Sensory nerve action potential

We collected the nerve conduction data of 46 normal people, including 58 hands, and calculated that there was a linear correlation between the SDL and the distance (D), as well as between the stimulation electrode and the recording electrode. We corrected MSDL (C-MSDL) and USDL (C-USDL) through the regression equation. We calculated the median nerve linear regression equation as C-MDSL = 0.954 + (0.01xD), F = 7.143, *P* < 0.05. C-USDL = 1.152 + (0.007xD), F = 4.215, *P* < 0.05.

### Statistical Analysis

SPSS25.0 was used to perform the Kolmogorov–Smirnov test to evaluate the normal distribution of the data. Parameters with a normal distribution were presented as the mean ± standard deviation (SD), and those with a non-normal distribution were expressed as the median values (M) and interquartile range (Q). Continuous variables that conformed to a normal distribution were evaluated using the analysis of variance, and variables with a non-normal distribution were compared using the Kruskal-Wallis H test. Normally and non-normally distributed data were analyzed with the Pearson correlation analysis and the Spearman correlation analysis, respectively. The receiver operating characteristic curve (ROC curve) was used to determine the validity of the diagnostic value. Statistical significance was determined at *P* < 0.05.

## Results

### Patient Characteristics

**Fig. 1:**
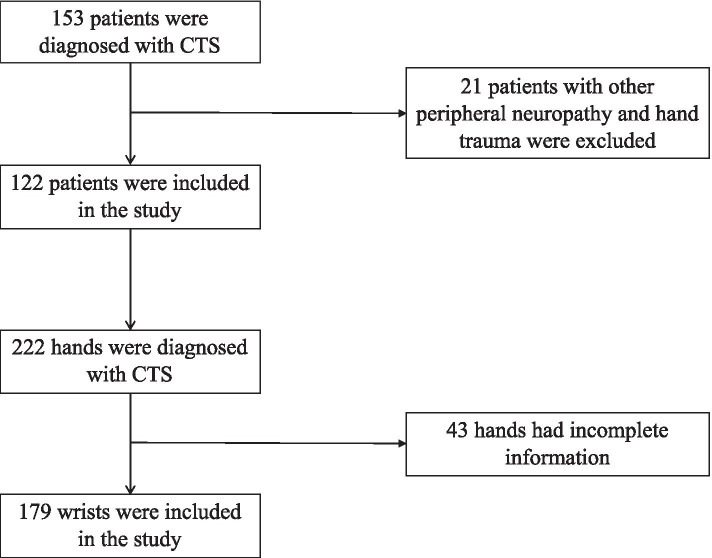
Flow chart of patient enrollment for the carpal tunnel syndrome (CTS) and control groups

A total of 122 patients with CTS met the inclusion criteria and were included in the study (Fig. 1). Twenty-two of the CTS patients were men (18.0%) and 100 were women (82.0%), with an average age of 54.43 ± 10.47 years (21–79 years). Among the total patient population, there were 100 patients (82.0%) with bilateral disease, 7 (5.7%) with CTS on the left hand, and 15 (12.3%) with CTS on the right hand, resulting in a total of 222 hands with CTS. Due to incomplete information for the ring finger MN or ulnar nerve (UN) latency, 43 hands were excluded, resulting in a total of 179 hands evaluated in the study. For the control group, electrophysiological data were recorded for 58 healthy wrists, comprising eight wrists for seven men and 50 wrists for 39 women, with an average age of 52.87 ± 13.42 years (17–77 years old).

The 179 CTS hands included in the study were divided into three groups based on the NCS results and CTS was classified as mild (109 hands, 60.9%), moderate (66 hands, 36.9%), and severe (4 hands, 2.2%). There were 17 patients with diabetes (27 hands), 4 patients with cerebrovascular accidents (6 hands), and 1 patient with rheumatoid disease (2 hands) (Table 2).

**Table 2:** List of clinical data for study patients

	Female	Male	Total
Average age (years)	54.69 ± 10.71	53.27 ± 9.42	54.43 ± 10.47
Number of patients	100 (82.0%)	22 (18.0%)	122
Total number of affected wrists	182 (82.0%)	40 (18.0%)	222
Number of wrists in the study	148 (82.7%)	31 (17.3%)	179
Number of cases on the right	11	4	15 (12.3%)^a^
Number of cases on the left	7	0	7 (5.7%)^a^
Number of bilateral cases	82	18	100 (82.0%)^a^
Number of hands in patients with diabetes	23	4	27
Number of hands in patients with rheumatoid disease	2	0	2
Number of hands in patients with cerebrovascular accident	5	1	6

^a^As a percentage of the total number of cases

### Comparison of CTS and Control using NCS and ROC Analysis

The Wilcoxon test indicated that MSDL and MUD in the CTS group were significantly higher than those in the control group (*P* < 0.01). There was no significant difference in ulnar nerve sensory action potential distal latency (USDL) between the two groups (*P* = 0.182) (Table 3).

**Table 3:** Comparison of SDL between CTS and control

Group	MSDL	USDL	MUD
Control (*n* = 52)	2.19 (0.10) ^a^	2.03 (0.07)	0.17 (0.03)
CTS (*n* = 179)	2.79 (0.45)	2.02 (0.27)	0.69 (0.45)
Z	−11.603	−1.067	−11.603
*P*	*P* < 0.01	*P* = 0.335	*P* < 0.01

*CTS* Carpal tunnel syndrome, *MSDL* Median nerve sensory action potential distal latency, *MUD* Median and ulnar sensory latency difference to ring finger, *SDL* Nerve sensory action potential distal latency, *USDL* Ulnar nerve sensory action potential distal latency

^a^ M (Q) indicates data not conforming to a normal distribution

**Fig. 2:**
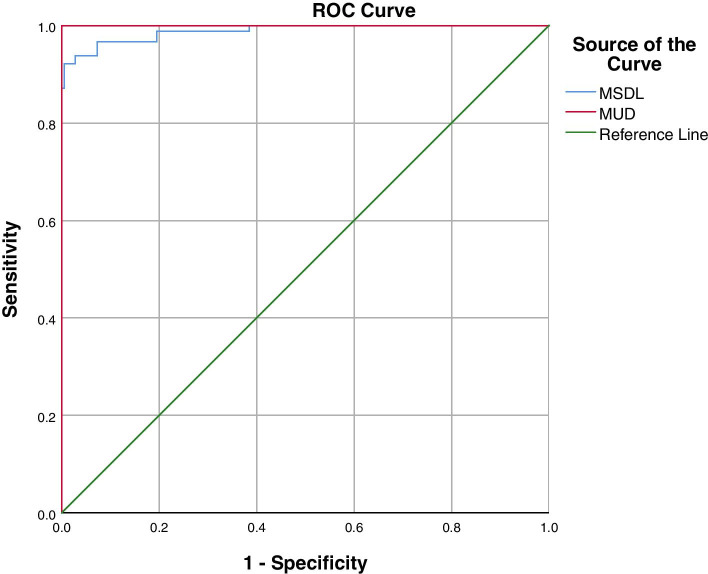
ROC curve of MSDL and MUD in CTS wrists. CTS, carpal tunnel syndrome; MSDL, median nerve sensory action potential distal latency; MUD, median and ulnar sensory latency difference to ring finger

As shown in the ROC curve in Fig. 2, the area under the receiver operating characteristic curve (AUC) of MSDL was 0.989 and the best cutoff value for diagnosing CTS was 2.40 ms, with a sensitivity of 92.2% and a specificity of 99.4%. When the AUC of MUD was 1, the best cutoff value for diagnosing CTS became 0.33 ms, with a sensitivity and specificity of 100%.

### Comparison and Correlation Analysis of NCS among CTS Severity Levels and Control

**Fig. 3:**
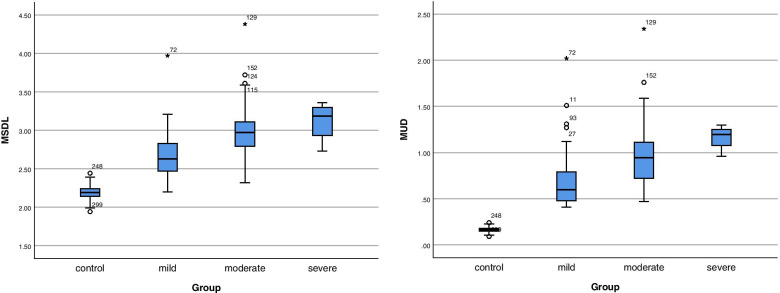
MSDL and MUD box plots for CTS severity

The CTS wrists were divided into 109 mild, 66 moderate, and 4 severe cases based on the NCS results. The Kruskal-Wallis H test indicated that there were significant differences in MSDL and MUD between the control and mild CTS groups, between the control and moderate CTS groups, between the control and severe CTS groups, and between the mild and moderate CTS groups (*P* < 0.01, respectively), but no significant differences between the mild and severe CTS groups (*P* = 0.66) or between the moderate and severe CTS groups (*P* = 1.00) (Table 4, Fig. 3).

**Table 4:** Ring finger sensory latency results in CTS and control groups

Group	MSDL	MUD
Control group	2.19 (0.10)	0.17 (0.03)
Mild CTS	2.63 (0.360)	0.60 (0.31)
Moderate CTS	2.97 (0.34)	0.945 (0.40)
Severe CTS	3.185 (0.49)	1.195 (0.26)
*P*	***P*** **< 0.01**^**abcd**^	***P*** **< 0.01**^**abcd**^

^a^*P* < 0.01 for mild CTS vs. control; ^b^*P* < 0.01 for moderate CTS vs. group; ^c^*P* < 0.01 for severe CTS vs control, ^d^*P* < 0.01 for mild vs. moderate CTS. *CTS* Carpal tunnel syndrome, *MSDL* Median nerve sensory action potential distal latency, *MUD* Median and ulnar sensory latency difference to ring finger

The data distribution shows that higher disease severity corresponded to greater MSDL and MUD. CTS, carpal tunnel syndrome; MSDL, median nerve sensory action potential distal latency; MUD, median and ulnar sensory latency difference to ring finger.

The Spearman correlation analysis shows a correlation between MSDL and CTS severity (*r* = 0.865, *P* < 0.01) and a correlation between MUD and CTS severity (*r* = 0.877, *P* < 0.01). In contrast, there was no correlation between USDL and CTS severity (*r* = −0.032, *P* = 0.548) (Table 5).

**Table 5:** Correlation analysis between SDL and CTS severity

	Severity	
	*r*	*P*
MSDL	0.865	**<0.01**
MUD	0.877	**<0.01**
USDL	−0.032	0.548

*MSDL* Median nerve sensory action potential distal latency, *MUD* Median and ulnar sensory latency difference to ring finger, *USDL* Ulnar nerve sensory action potential distal latency

## Discussion

In this study, the average age of patients with CTS was 54.43 ± 10.47 years, with a male to female ratio of about 1/4.5. This larger proportion of female patients compared to male patients is similar to that in previous studies [1, 12, 13] and may be caused by a combination of factors such as hormonal changes in women and performing more housework in the family. Consistent with previous findings [12, 14, 15], bilateral incidence had the highest occurrence (82%) among patients, and the number of patients with CTS on the right hand was higher than those with the disorder on the left hand. The greater incidence on the right side may be because most people are right-handed and perform more repetitive movements using the right hand than the left hand.

Some studies have shown that patients with CTS may have UN damage [16, 17]. A study by Kang et al. found that when patients with CTS underwent carpal tunnel release, the pressure in Guyon’s canal was reduced and the sensory conduction of the UN improved [16]. However, in several studies with larger patient populations, UN conduction was found to be unaffected in patients with CTS [18–20]. In our study, a large number of wrists affected by CTS (179) were included, and no significant differences in the USDL were found between the CTS and control groups or between groups with different levels of CTS severity, suggesting that the UN was not significantly damaged in the patients with CTS.

Our study showed that the MSDL and MUD of the patients with CTS were significantly different from those of the control group, with high diagnostic accuracy, evidenced by a value of 1 for the AUC of MUD and 0.989 for the AUC of MSDL. The higher MUD AUC compared to the MSDL AUC indicates that MUD is more accurate than MSDL in the diagnosis of CTS, which is consistent with the results of many studies reported thus far [2, 3, 21, 22]. This difference may be due to individual differences, such as age, gender, weight, and workload that result in different individual neurological states. For example, neurological function often declines in older patients, leading to relatively larger MSDL and USDL measurement values. A simple increase in MSDL does not indicate that CTS should be diagnosed, but a comparison with the UN on the same hand and MUD calculation can provide better insight regarding the problem. Most of the previously reported MSDL cut-off values were between 2.7 and 3.8 ms (mostly around 3.7 ms), with a sensitivity of 67% ~ 90% and a specificity mostly above 90% [3, 14, 15, 21, 23–26]. In our study, the sensitivity of MSDL for the diagnosis of CTS was 92.2% and the specificity was 99.4%, which were similar to the results of previous studies.

On the other hand, we found that the optimal cut-off value for MSDL was 2.40 ms, which was smaller than the previously reported results. This could be because 97.7% of patients in this study had mild and moderate CTS, which were far more than the proportion of patients with severe CTS and led to a lower MSDL value. Our study showed that CTS can be diagnosed with a lower MSDL, in contrast to the current belief that the critical value for MSDL is 4 ms. The best cutoff value for MUD to diagnose CTS was 0.33 ms, with a sensitivity and specificity of 100%. Previous studies found that the diagnostic cutoff value was about 0.35 ~ 0.81 ms with a sensitivity of 85% ~ 90% and a specificity of 85% ~ 96.7% [3, 14, 21, 23, 27, 28]. The optimal cutoff value of MUD for diagnosing CTS was 0.33 ms in the present study, which is consistent with the results of previous studies. The difference is that the sensitivity (100%) and specificity (100%) in this study were higher than those in previous studies, possibly because the control consisted of the contralateral hand of patients with unilaterally affected wrist, which provided good comparability.

There were significant differences in the MSDL between different CTS severity groups (mild, moderate, or severe) and the control group and between mild and moderate CTS. Higher MSDL corresponded to greater severity. Correlation analysis showed a positive correlation between MSDL and CTS severity (rs = 0.865, rs > 0), indicating that the severity of CTS increases as the MSDL increases. Similarly, there were significant differences in MUD between the different degrees of CTS severity and the control and between mild and moderate CTS. As shown in the box plot (Fig. 3B), higher MUD corresponded to greater severity. Correlation analysis showed a positive correlation between MUD and severity. The correlation coefficient of MUD (rs = 0.877) was greater than that of MSDL (rs = 0.865), indicating that the correlation of MUD to CTS severity was better than that of MSDL. This is consistent with our previous finding of MUD AUC > MSDL AUC. MUD was found to be more accurate than MSDL in diagnosing CTS.

Similar to previous studies, our study was conducted using the CTS electrophysiological severity grading method described by Padua et al. [29]. The complete MN motor conduction and the sensory conduction are needed to distinguish CTS severity. We found that MUD was correlated with the severity of CTS. For patients who are more sensitive to pain and cannot tolerate electrical stimulation, perhaps MUD measurement alone can accurately reflect the CTS severity and minimize the pain experienced by the patient. The information can further guide the patient to choose an appropriate treatment plan.

### Limitations

Due to the low number of wrists with severe CTS, the results of this study may not be applicable to all degrees of CTS severity. However, this study included enough patients with mild and moderate CTS, and thus the electrodiagnosis still have great diagnostic value. More patients with severe CTS may need to be included to determine the optimal cutoff values for MUD to classify CTS in the future.

## Data Availability

The datasets used and/or analysed during the current study available from the corresponding author on reasonable request.

## Abbreviations

AUC: Area under the receiver operating characteristic curve
CMAP: Compound muscle action potential
CTS: Carpal tunnel syndrome
EDX: Electrodiagnostic
M: Median value
MN: Median nerve
MSDL: Median nerve sensory action potential distal latency
MUD: Median and ulnar sensory latency difference to ring finger
NCS: Nerve conduction studies
Q: Interquartile range
ROC: The receiver operating characteristic curve
SD: Standard deviation
SDL: Nerve sensory action potential distal latency
SNAP: Sensory nerve action potential
UN: Ulnar nerve
USDL: Ulnar nerve sensory action potential distal latency
